# Genome-wide profiling of long non-coding RNAs from tomato and a comparison with mRNAs associated with the regulation of fruit ripening

**DOI:** 10.1186/s12870-018-1300-y

**Published:** 2018-05-04

**Authors:** Minghui Wang, Weihua Zhao, Lei Gao, Lingxia Zhao

**Affiliations:** 1 BRC Bioinformatics Facility Cornell University, Ithaca, 14850 USA; 2 Joint Tomato Research Institute, School of Agriculture and Biology, Shanghai Jiao Tong University, Shanghai, 200240 China; 3 Plant Biotechnology Research Center, School of Agriculture and Biology, Shanghai Jiao Tong University, Shanghai, 200240 China

**Keywords:** Tomato, LncRNAs, DNA methylation, Ripening

## Abstract

**Background:**

Long non-coding RNAs (lncRNAs) are involved in multiple biological processes in both mammals and plants. There is growing evidence that they are associated with development; but their expression and regulation during fruit ripening in the model plant tomato (*Solanum lycopersicum*) has yet to be described.

**Results:**

Following integration of 134 RNA-seq data sets, we identified 79,322 putative lncRNAs, consisting of 70,635 lincRNAs, 8085 antisense non-coding RNAs (ancRNAs) and 602 sense lncRNAs (slncRNAs). lncRNAs had specific features that are distinct from mRNAs, including tissue-specificity, and shorter and fewer exons. Notably, more than 5000 of the novel lincRNAs were found to be expressed across the mature green (MG), breaker (BR) and breaker plus 7 days (BR + 7) developmental stages. The differently expressed lincRNAs had different DNA methylation profiles from the mRNAs.

**Conclusions:**

Integrating transcriptome datasets and genome-wide screening enabled the identification of a comprehensive set of tomato lncRNAs. Here, we found that the lncRNAs DNA methylation profiles were different from those of mRNAs. This will help future investigation of lncRNA function, especially for the dissection of the molecular mechanisms involved in the regulation of fruit development.

**Electronic supplementary material:**

The online version of this article (10.1186/s12870-018-1300-y) contains supplementary material, which is available to authorized users.

## Background

Tomato (*Solanum lycopersicum*) is a major vegetable crop worldwide [1], and an important model species for studying the development and ripening of fleshy fruits [2–6]. Tomato development is a complex biological process, involving tightly regulated cell division and differentiation, cell expansion, and finally ripening [7]. During this ripening phase, the coordinated activation of multiple genes and regulatory pathways result in changes in color, flavor, aroma, texture and nutritional attributes [8, 9]. Many technologies and resources have been developed to study tomato ripening and to characterize processes and mechanisms that control fruit development [5, 10]. Such studies have revealed transcription factors that affect ripening [9, 11, 12], key ripening related genes and gene networks underlying ripening mutants [13–16], as well as genes that related to hormone ethylene or DNA demethylation [17–19]. However, the identities of upstream regulators involved in tomato fruit ripening, such as non-coding RNAs (ncRNAs), have yet to be elucidated in detail.

Non-coding RNAs (ncRNAs), which do not encode proteins, comprise a substantial portion of eukaryotic transcripts [20, 21]. They can be divided into various classes, including microRNAs (miRNAs), piwi-interacting RNAs (piRNAs), lncRNAs, and others with a length of more than 200 nucleotides [22]. In plants, these regulators mainly participate in biological processes that coordinate gene expression, particularly during development [23]. The functions of miRNAs have been widely studied in the context of their involvement in regulating translation or in directing the degradation of specific mRNA targets [24–27]. Indeed, numerous studies have highlighted the importance of miRNAs in regulating a wide range of plant developmental processes, including the formation of seeds, fruit and other organs [28–30]. There is also evidence that other classes of ncRNAs contribute to developmental regulation of plants [31], however, the range of functions for several classes, such as the large and diverse populations of lncRNA, is not well characterized in plants.

LncRNAs can be divided into four main categories, based on their genomic location relative to protein coding genes: (i) long intergenic ncRNAs (lincRNAs), which are non-overlapping with the exons of protein-coding genes; (ii) intron ncRNAs, which are situated in intron regions; (iii) antisense ncRNAs (ancRNAs), which are transcribed from the complementary DNA strand such that they have potential to pair to the mature mRNA [32], and (iv) sense ncRNAs (slncRNAs), which are defined as overlapping with the sense strand of protein-coding genes. Only a few lncRNAs have been reported to regulate plant development [33], but functional analyses have shown that they are potent *cis*-or *trans*-regulators that influence the transcriptional activity of their target loci [34]. LncRNAs have also been shown to modulate genome activity and control chromatin remodeling [35], and they exhibit notable tissue-specific expression patterns and relatively lower expression levels, compared to mRNAs [33, 36–38].

Next-generation sequencing (e.g. RNA-seq) has driven genome-wide discovery and analysis of non-coding RNAs in plants, including maize (*Zea mays*) [39], rice (*Oryza sativa*) [33], *Arabidopsis thaliana* [40], among others. For example, strand-specific paired-end RNA sequencing was used to identify 1565 lncRNAs involved in yellow leaf curl virus (TYLCV) infection by virus-induced gene silencing (VIGS) [41], and to detect 3679 lncRNAs from wild-type tomato and ripening mutant fruit [42]. Wang et al. (2016) identified 413 and 709 multi-exon lncRNAs in the tomato Heinz1706 cultivar and its wild relative LA1589, respectively [43], and Cui et al. (2017) found that lncRNAs played important roles in the response to infection in tomato by comparative transcriptome analysis [44]. However, these studies included only a few samples and were largely limited to a certain developmental stage or tissue, from which it can be inferred that only subsets of the tomato lncRNAs have been reported. Compared to the more than 6000 intergenic lncRNAs and 37,238 ancRNA pairs identified in one *A. thaliana* study [45], we hypothesized that the lncRNA annotation of tomato is far from complete. Moreover, lncRNA expression patterns differ not just between tissues, but also change across different development stages.

A comprehensive map of the entire set of lncRNAs present in the tomato genome would require a comprehensive study of the tomato transcriptome. In this study, we used 14 non-strand-specific tomato RNA-seq datasets derived from the MG, BR and BR + 7 stages of fruit development. Combined with 120 publicly available tomato RNA-seq datasets corresponding to a variety of organs and developmental stages, we generated 43 non-strand-specific RNA-seq and 91 strand-specific RNA-seq datasets. We included datasets from both cultivated tomato (*S. lycopersicum*) and its wild relative (*S. pimpinellifolium*). Our results suggest that lncRNAs exhibit tissue-specific expression patterns in tomato, and have low expression and short length compared to mRNAs. We describe the identification of candidate lncRNAs that are likely to have roles in tomato ripening regulation, and compare the expression patterns and DNA methylation dynamics between lncRNAs and mRNAs.

## Results

### Identification of tomato lncRNAs

**Fig. 1:**
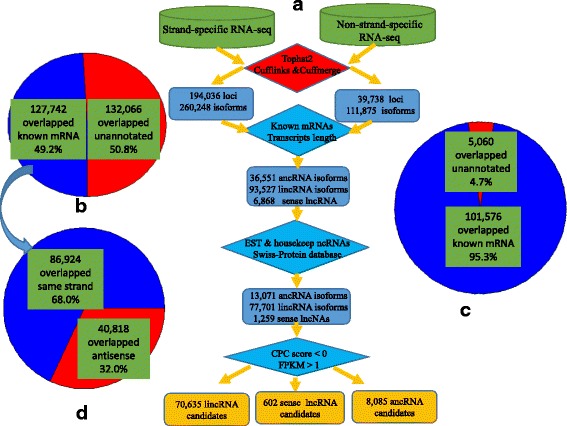
Identification of long noncoding RNAs in tomato. **a** The pipeline for identification of lncRNAs in tomato. **b** The number of novel isoforms and their relative proportion in the strand specific data set. **c** The number of novel isoforms and their relative proportion in the non-strand specific data set. **d** The number of novel isoforms and the relative proportion of ancRNAs

A total of 134 high-throughput sequencing data sets derived from more than 10 different organs (see Additional file 1) were used to identify lncRNAs. An overview of the analysis pipeline is shown in Fig. 1a. Using this pipeline, we produced a set of 194,036 expressed loci with 260,248 transcript isoforms in the strand-specific database, and 39,738 expressed loci with 111,875 transcript isoforms in the non-strand database. Comparing with ITAG annotation file (ITAG 2.4) comprising 34,725 protein-encoding transcripts, 132,066 transcript isoforms (50.8% of total isoforms) were identified from unannotated regions in the strand-specific dataset (Fig. 1b), while 5060 transcript isoforms were found in the non-strand specific dataset, accounting for 4.7% of the total number of isoforms (Fig. 1c). These transcript isoforms are mainly transcribed from unannotated regions of the genome, and are referred to here as lincRNAs. Only the transcripts with strand assignments were considered in the subsequent analyses. We found that the transcripts overlapping with known mRNAs from the complementary DNA, namely ancRNAs, corresponded to approximately 32% of the total 127,724 Fig. 1d). Only 6895 potential lncRNAs overlapped with the sense strand and no intron located lncRNAs were recovered from our pipeline. Both lincRNAs, ancRNAs and slncRNAs were used as the starting point to predict lncRNA candidates in tomato. To ensure that unannotated transcripts were not missed due to mapping artifacts or transcriptional noise, stringent filtering steps were applied (see Methods section). Finally, we identified 79,322 expressed lncRNAs, of which 70,635 (89.0%) were lincRNAs, 8085 (10.2%) were ancRNAs and 602 (0.8%) were slncRNAs (Additional files 2, 3 and 4).

### Characterization of tomato lncRNAs and their relationship with TEs

**Fig. 2:**
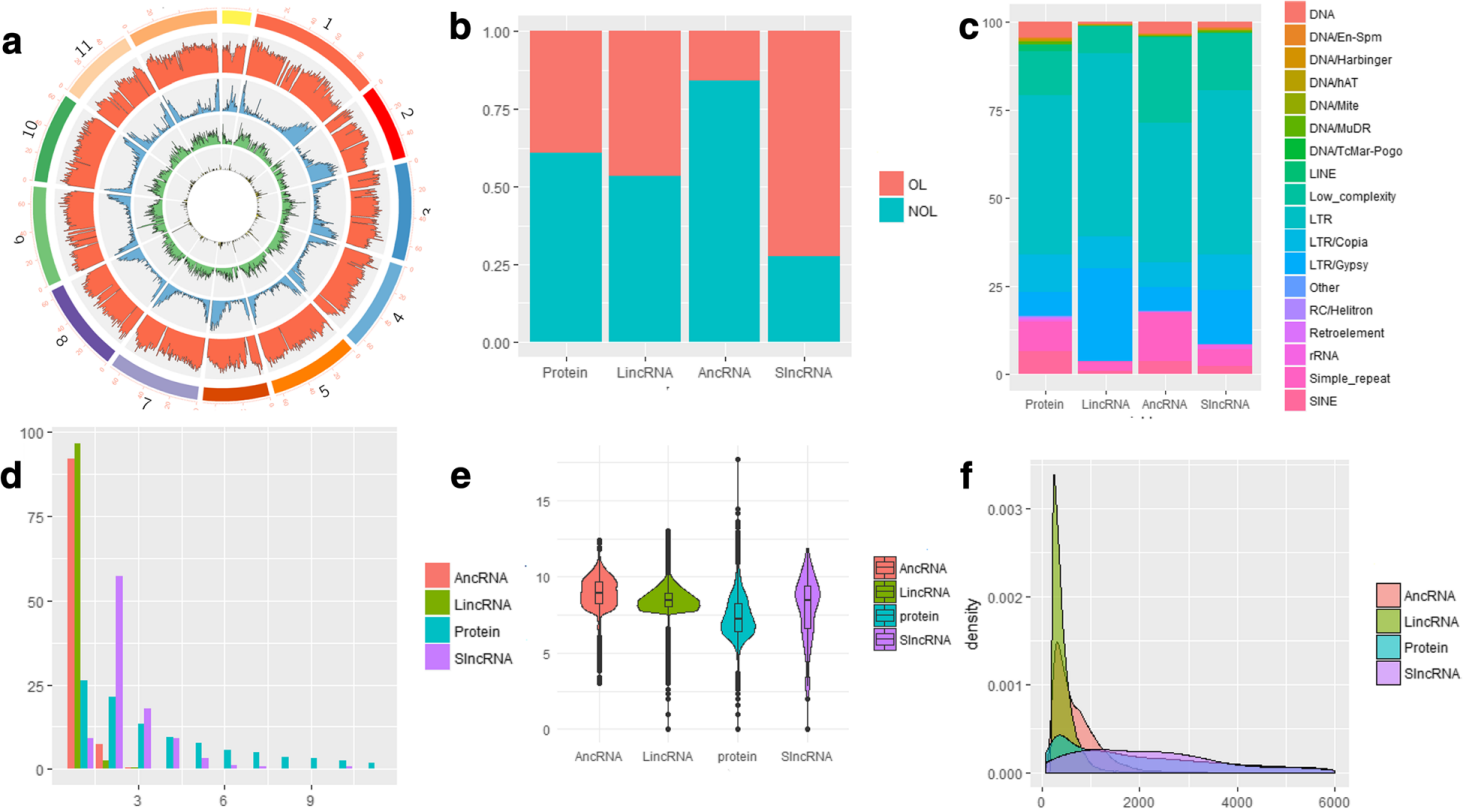
Comparison of lncRNA and mRNA characteristics. **a** Distribution of transposable elements (TE, orange), mRNAs (blue), lincRNAs (green) and ancRNA (yellow) on each chromosome. **b** The number of ancRNA, lincRNA, slncRNAs and mRNAs overlapping with TEs; overlapping in orange and non-overlapping in turquoise. **c** The proportion of different TE types overlapping with lncRNAs and mRNAs. **d** Number of exons in lncRNAs and mRNAs. **e** lncRNA and mRNA exon lengths. **f** Compassion of the length of lncRNAs and mRNAs

Like most plants species, the tomato genome contains a lot of repetitive DNA; specifically 459 million base pairs (Mb), which corresponds to ∼55.7% of the genome. We found that LTR-retrotransposon copies were the most abundant transposable elements (TEs), covering 78.2%, while Gypsy- and Copia-type elements representing 20.0 and 9.6%, respectively. Class II elements (DNA transposons) represent only a minor fraction of the repetitive DNA in tomato. A sliding window density analysis showed that the lincRNAs are fairly evenly distributed across the chromosomes relative to mRNAs (Fig. 2a), and a similar pattern was observed for repeat components (Fig. 2a). Protein coding transcripts were found to be mainly located in the distal chromosome arms, with a lower density around the pericentromeric regions. LincRNAs and ancRNAs showed many common features, but also minor differences (Fig. 2). 46.8% lincRNAs and 15.9% of ancRNAs overlapped with repetitive sequences, while mRNAs are only account for 39.1% (Fig. 2b). The lincRNAs were enriched in LTR (Fisher’s Exact, *P* < 2.2e-16) and LTR/Gypsy (Fisher’s Exact, P < 2.2e-16) regions compared to mRNA (Fig. 2c), while the ancRNAs overlapped more with low complexity (Fisher’s Exact, P < 2.2e-16) and simple repeat (Fisher’s Exact, P < 2.2e-16). Some lncRNAs were found to have more exons than mRNAs (Fig. 2d), but most (91.9%) contain a single exon that is longer than mRNA exons (Fig. 2e). The median exon lengths are: ancRNA = 497, lincRNA = 351, and mRNAs = 148 (Mann-Withney U test; P < 2.2e-16). Most of the lncRNAs (98%) are less than 2000 bp long (Fig. 2f), which is shorter than the 2047 bp median value for mRNAs.

### Tissue specific expression patterns

**Fig. 3:**
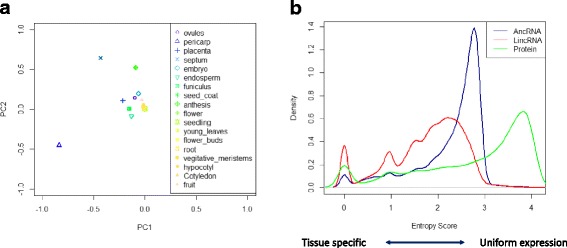
Tissue-specific expression analysis of lncRNAs and mRNAs. **a** Principal component analysis (PCA) was performed of data from different tissues obtained using different sequencing platforms. **b** Density plot showing the entropy score of the lncRNAs and mRNAs. The entropy score has bit units ranging from zero for genes expressed in a single tissue to log2 of the number of tissues for genes expressed uniformly in all tissues considered

We used principal component analysis (PCA) to investigate the main sources of variation in gene expression, based on data derived from 18 tomato organs. A scatter plot of the PCA showed that the top 2 principal components comprise 80% of the variance (Fig. 3a) and that there was no pronounced dominating effect due the use of different sequencing platforms. Only two data sets, derived from septum and pericarp organs, show large variation compared to the others, and we concluded that these differences are primarily associated with the specific characters of the tissues and not the sequencing platform.

Entropy values were used to evaluate lncRNA tissue specific expression patterns, with the result being defined as tissue-specific, heterogeneous, or uniformly expressed, based on an arbitrary entropy score cutoff value, ranging from 0 (transcripts expressed only in one tissue) to 4.17 (genes having the same expression level across all tissues). AncRNAs mainly belonged to the heterogeneous category (89.2%), with a score between 1 and 2 (Fig. 3b). For the lincRNAs the tissue-specific and heterogeneous scores were 19.3 and 79.5%, respectively, while 54.2% of the mRNAs were assigned to the uniform expression category (score > 3), and only 13% to the tissue-specific category. Overall, both the lincRNA and ancRNA entropy scores were far more uniformly distributed compared with the mRNA scores (Kolmogorov-Smirnov test, *P* < 2.2e-16).

### lncRNAs with potential function in tomato fruit ripening

**Fig. 4:**
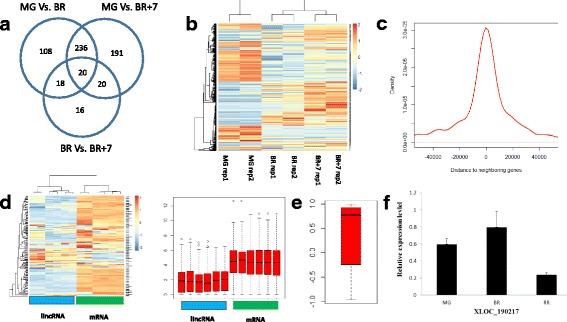
Differently expressed lncRNAs involved in the ripening process. **a** Venn diagram showing differently expressed lncRNAs in three different comparisons, MG (mature green), BR (breaker) and (BR + 7) breaker plus 7 days. **b** Heatmap showing the normalized read counts of differently expressed lncRNAs across three development stages. **c** The distance distribution of differently expressed lncRNAs to nearby mRNAs. **d** Heatmap and box plot showing the expression level of lincRNA-mRNA pairs. **e** Pearson correlation distribution of lincRNA-mRNA pairs. **f** Real-time (RT)-PCR validation of lincRNA expression

LncRNAs with an average FPKM value > 10 were considered to play a role in the ripening process, and 4079 were identified in the MG stage, 4135 in the BR and stage and 4311 in the BR + 7 days stage (Additional file 5). Next, we compared the temporal changes in lncRNA expression using a cutoff value of |log2(FC)| > 1 and FDR < 0.05. The Venn diagram in Fig. 4a shows the number of shared and exclusive differentially expressed (DE) lincRNAs between different developmental stages. Only 20 (3.3%) of the DE lincRNAs were expressed in all three development stages, while 108 (17.7%) were DE exclusively between the MG and BR stages, 191 (31.4%) between the MG and BR + 7 stages, and 16 (2.6%) between the BR vs BR + 7 stages. Additionally, 97.4% developmentally regulated lincRNAs showed changes in their levels of expression between the MG and BR, or MG versus BR + 7 stages, and 42.1% of the differently expressed lincRNAs were shared between the BR and BR + 7 versus MG stages (Additional files 6, 7, 8 and Fig. 4a). These numbers are lower than for the differently expressed mRNAs, where 99.3% changed between MG and BR, and MG versus BR + 7, and 62.4% shared between MG and BR and MG versus BR + 7.

Next, we examined the kinetics of lincRNA expression, as shown in Fig. 4b as a heatmap of normalized read counts. We found that changes occurred mainly at early or late developmental stages. When the relative distance and correlation of DE lincRNAs with the nearest DE mRNA genes was analyzed, we observed that most (71.3%) of lincRNAs were in closer proximity to the mRNAs with a distance less than 30 kb (Fig. 4c). In subsequent analyses, we focused on the subset of lincRNAs that were within 10 kb of mRNAs, corresponding to a total of 95 lincRNA-mRNA pairs (Additional file 9) that were significantly DE across developmental stages. The heatmap and boxplot figures in Fig. 4 show that the lincRNAs genes were expressed at lower levels than the nearby mRNAs (Fig. 4d). Large proportion of pairs with a positive Pearson correlation and a medium value ≥0.77 (Fig. 4e), are likely co-transcribed during ripening. To confirm this, the top 9 lincRNAs with positive correlation with mRNAs were selected for real-time quantitative PCR (qRT-PCR) verification (primer information and results shown in Additional file 10). Of the 9 lincRNAs, 7 exhibited consistent expression patterns (up- or downregulation at the same development stage) based on the qRT-PCR results and the RNA-seq data (Fig. 4f & Additional file 10), validating the co-transcription. Individual inspection showed that some of the lncRNAs were associated with ripening. For example, we identified a lincRNA (XLOC_055641, Chr3:64108555–64,110,184), adjacent to Solyc06g051800, which was specifically expressed in ripening fruit. *Solyc06g051800* has also been reported to be a target of ethylene regulation [46]. The expression of *XLOC_055641* and *Solyc06g051800* both increased (> 4 fold) from the unripe to ripe stage of fruit development, suggesting a relationship between their respective functions and/or regulation. The function of most co-regulated lincRNAs is not known, and they are a target for further research.

### LncRNAs and mRNAs with different DNA methylation levels

**Fig. 5:**
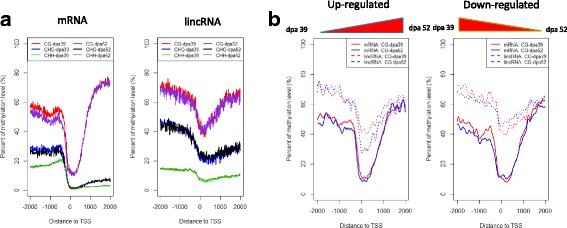
DNA methylation levels near transcription start sites. **a** DNA methylation level distribution in mRNA and lincRNA around the transcription start site (TSS) region, including CG, CHG and CHH methylation. mRNA is shown on the left and lincRNA on the right. **b** CG methylation level changes in up-regulated (log2 (dpa 52/dpa 39) > 1) and down-regulated (log2 (dpa 29/dpa 52) > 1) in mRNAs and lincRNAs

DNA methylation is more likely to be associated with promoter regulatory regions [47]. To determine differences in promotor methylation levels between expressed mRNAs and lincRNAs (FPKM > 10), the average methylation signal within a 2 kb region around the transcriptional start site (TSS) was examined. The lincRNAs displayed a relatively higher CG and CHG methylation density than the mRNAs (Fig. 5a). Compared to the lincRNAs, the mRNA CHG and CHH profiles had a more pronounced decrease immediately downstream of the TSS, whereas CG methylation rose sharply (Fig. 5a). Although the CG methylation density was also reduced near the TSS for the lincRNAs, it still remained approximately 4-fold higher, than for the mRNAs (Fig. 5a). Further exploration of the pattern across different development (39 days post-anthesis [dpa] versus 52 dpa) stages showed that mRNAs from the late developmental stage (52 dpa) had significantly lower CG and CHG methylation levels upstream of the TSS than the early developmental stage (39 dpa) (Kolmogorov-Smirnov test, *P* < 2.2e-16 for CG and CHG). For the lincRNAs, a significant difference was only seen for CG methylation levels (Kolmogorov-Smirnov test, *P* = 1.986e-05). This indicates that most lincRNAs have identical or stable CHG and CHH methylation patterns to their host genes across fruit development. Next, the relationship between variation in expression and CG methylation levels was investigated from the unripe to the ripe stage. We assumed that reduced expression (down-regulation) indicates hypermethylation (silencing), and increased expression was related to hypomethylation (activation) [48]. We identified sets of genes that were up- or down-regulated from the unripe to the ripe stage with a |log2FC| > 1. We detected an inverse correlation between an increase in CG methylation levels and expression of the ripening related mRNAs and lincRNA genes, so that the mRNA median CG value went from 47.7 at 39 dpa down to 44.4 at 52 dpa, and the lincRNA median CG value went from 61.9 at 39 dpa to 58.5 at 52 dpa in the up-regulated group (Fig. 5b). Lastly, mRNAs and lincRNAs showed significant differences in CG methylation levels (Kolmogorov-Smirnov test, *P* < 0.001), especially in the down-regulated group. Taken together, the lincRNAs and mRNAs had significantly different DNA methylation patterns (P < 2.2e-16 for Kolmogorov-Smirnov test).

## Discussion

To identify a comprehensive set of tomato lncRNAs, we integrated de novo generated and previously published RNA-seq data sets (Additional file 1). Similar analyses have been performed using model plants, such as *A. thaliana* [38, 45], rice [33], maize [39] and wheat (*Triticum aestivum*) [49]. Other reports have described the regulation of lncRNA in tomato [42, 44]; however, we hypothesized that these only identified a subset of lncRNAs as relatively few samples were considered. Moreover, the coverage of the RNA-seq analysis also influences the identification of transcripts, such that a higher sequencing depth would result in the identification of genes that are expressed at lower levels [50]. To this end, we produced high coverage RNA-seq data and combined it with publicly available data, including data from a diverse set of organs, to reveal a more complete set of tomato lncRNAs. This resulted in the identification of a significantly higher number of transcripts than previous studies [42]. Using a similar pipeline to a maize study [39], we identified more than 78,720 tomato lncRNAs, and based on a recent genome annotation release (ITAG2.4), > 95% of mRNA sequences were recovered here, suggesting that the lncRNAs identified by this pipeline were correctly identified. Some of the large number of putative lncRNAs may be non-coding transcripts expressed in different organs and developmental stages, and additionally, it is difficult to exclude the influence from non-polyadenylated transcripts. For example, the strand-specific data from one study [51], omitted the polyA isolation step during library preparation, and some of the identified lncRNA may therefore represent non-polyadenylated transcripts. Recently, novel non-polyadenylated transcripts were reported from rice and *A. thaliana* [52, 53], which had a low protein-coding potential. In this study, we compared different features between lncRNAs and mRNAs, and found that lncRNAs are relatively short, have a low number of exons, and have relatively specific tissue-specific expression. lncRNAs have no protein encoding potential, but exert a regulatory function that may be related to a small RNA dependent mechanism [54]. Additionally, in plant genomes, a distinctive pathway has been reported that utilizes noncoding RNA for epigenetic regulation [54], suggesting that lncRNAs may be involved in RNA regulation.

As part of an examination of lncRNAs associated with the regulation of tomato fruit ripening, we identified nearly 5270 that were highly expressed across the MG, BR and BR + 7 fruit development stages, and 467 lncRNA that appeared to be differently expressed between the MG and BR stages. To further resolve the potential functions of these ripening related lncRNAs, we focused on co-regulated lncRNA/mRNA pairs, with the aim of identifying candidates for further study. Most of the co-regulated mRNAs were not functionally annotated, and will require further investigation to elucidate the mechanism by which they may influence ripening.

DNA methylation plays a crucial role in plant development, including gametogenesis, seed development, and response to stresses [55–57]. and lncRNAs are also important regulators of many biological processes [58]. However, the association of epigenetic/epigenomic features with lncRNAs has not been studied in tomato. By characterizing the genome wide DNA methylation profile across the MG and BR developmental stages, we showed that the DNA methylation level varies between lincRNAs and mRNAs. Besides differences in the pattern, mRNAs show dynamic changes in DNA methylation that are relatively low in the later stages of development. Only the CG density in lncRNAs showed a similar pattern to that of mRNAs, but still had a 4 fold higher density, while CHG and CHH methylation was relatively stable across development. This may cause the repression of lncRNAs and maintenance of genome stability. Finally, while both lncRNAs and mRNAs had high DNA methylation levels in down-regulated genes and low levels in up-regulated genes during the ripening process, we observed higher CG methylation levels in the lincRNAs compared with the mRNAs. This may be because lncRNAs are very important during development, and so their regulation by DNA methylation is relatively stable, or there may be other factors associated with lncRNA regulation that ensure functional stability.

## Conclusions

In this study, we integrated 134 data sets and identified 79,322 lncRNAs, including lincRNAs, ancRNAs and slncRNAs. Compared with mRNAs, the lncRNAs were relatively short, had fewer exons and a very tightly controlled tissue-specific expression. Moreover, changes in expression were identified in lincRNAs across developmental stages (MG, BR and BR + 7) implying that they are involved in the regulation of ripening. lncRNAs were observed to have different DNA methylation patterns than mRNAs. The comprehensive annotation of tomato lncRNAs presented here provides a valuable resource for further functional studies.

## Methods

### Data sources

A total of 91 strand-specific and 29 non-strand-specific RNA-seq datasets (Additional file 1) were obtained from the Gene Expression Omnibus (GEO) of the National Center for Biotechnology Information (NCBI; https://www.ncbi.nlm.nih.gov). Fourteen additional non-strand-specific datasets, including data from the MG, BR and BR + 7 fruit stages, were produced by our group. The public RNA-seq data were obtained from more than 10 different organs (leaf, seeds, peel, seed, root, flesh, embryo, anther, buds, hypocotyls) encompassing a total of 3.5 billion reads with read lengths ranging from 51 to 251 nucleotides. The publicly available tomato EST database (https://www.sgn.cornell.edu), the plant snoRNA database (http://bioinf.scri.sari.ac.uk/cgi-bin/plant_snorna/home) and the tomato sRNA database (http://ted.bti.cornell.edu/cgi-bin/TFGD/sRNA) were used for filtering potential lncRNAs.

### Bioinformatics pipeline for identifying lncRNAs

Ribosomal RNA sequences (rRNA) were first removed from the raw sequence reads using the SILVA database (http://www.arb-silva.de) and then aligned to the tomato genome assembly build 2.50 and annotation ITAG2.4 (http://solgenomics.net/organism/Solanum_lycopersicum/genome) using the spliced read aligner TopHat (version 2.1.0) [59]. For the strand-specific data, the parameters were set as: tophat2 -num-threads ‘14’ --library-type ‘fr-firststrand’ splice-mismatches ‘0’ -min-intron-length ‘70’. For the non-strand specific data, the parameters were: tophat2 -num-threads ‘14’ splice-mismatches ‘0’ -min-intron-length ‘70’. The transcriptome identified in each experiment was separately assembled using Cufflinks (version 2.2.1) [60]. Transcripts from each library with a FPKM > 1 were combined using Cuffmerge and two separate transcripts lists were generated.

We applied multiple filtering procedures to remove potential artifacts. First, for lincRNAs, the transcripts overlapping once with the reference annotation were removed; for ancRNAs, only the transcripts overlapping with the reference on the opposite strand were kept; for sense ncRNAs, the transcripts with class code ‘o’ were kept; for intron lncRNAs, transcripts falling entirely within a reference intron were kept. Second, transcripts shorter than 200 bp were removed. Third, tomato EST database, the plant snoRNA database and the tomato sRNA database were used to filter the data set by BLASTN (EST filtering: E-value cutoff 1e-10, alignment coverage > 80%, and identity > 80%; housekeeping ncRNAs: E-value cutoff 1e-10, identity > 90% and minimized alignment length > 22 bp). Fourth, the Swiss-Protein database was used to eliminate putative protein-encoding transcripts using BLASTX (E-value cutoff 1e-10, alignment length > 50 bp). Additionally, transcripts with a Coding Potential Calculator (CPC) > 0, based on a support vector machine theory, were removed. Finally, FPKM values were also considered across samples, and only transcripts with a FPKM > 1 in at least one sample were kept.

### Repetitive element analysis of lncRNAs

An annotation for the tomato genome (genome assembly build 2.50) was downloaded from the SOL database (https://solgenomics.net/organism/Solanum_lycopersicum/genome). The BEDTools intersect command was used to identify overlap between repetitive elements and lncRNAs [61]. Repetitive elements that overlapped with at least 10 nucleotides with lncRNA were considered for further analysis.

### Distribution of lincRNAs and repetitive elements

The distribution of lncRNAs, including lincRNAs and ancRNAs, and repetitive elements was determined using Circos with a sliding window size of 500 kb.

### Assessing tissue-specific characteristics of lncRNAs

In order to identify tissue-specific characteristics of the lncRNAs, relative frequencies of tissue expression levels (FPKM), *p*_*ij*_ were calculated; for the ith gene (*i* = 1, 2,…, g) in the jth tissue (j = 1, 2,…, t). Shannon’s entropy formula was directly applied to measuring tissue specificity characters.

$$ Hi=-\sum \limits_{n=1}^t\left({P}_{ij}\ast {logP}_{ij}\right) $$

Entropy (H) measures the degree of overall tissue specificity of a gene. H ranges from zero to log2 (N), with the value 0 for genes expressed in a single tissue and log2 (N) for genes expressed uniformly in all the tissues. We therefore relied on the entropy score distribution to measure tissue-specific expression patterns for lncRNAs.

### Processing of whole-genome bisulfite sequencing data

The whole-genome bisulfite sequencing data sets derived from 39 dpa and 52 dpa fruit were retrieved from the SRA database (SRA046092). Raw sequence data were mapped to the tomato genome assembly build 2.50 using Bismark 0.16.3 [62] with the Bowtie2 (version 2.2.8) alignment tool [63]. The percent methylation of cytosines in a CpG, CHG or CHH context was calculated by the proportion of 100* (methylated reads)/ (methylated reads + unmethylated reads). The average DNA methylation level was calculated within 2 kb region around the transcript start site (TSS).

### Real-time quantitative (RT-PCR) verification of lincRNAs

To verify the co-regulated lincRNAs/mRNAs, RNA was extracted from *S. lycopersicum* cv.M82 (provided by Professor J. Rose, Cornell University, USA), which is the same cultivar used for the MG, BR and BR + 7 RNA-seq data. A set of 9 transcripts with the highest correlation of lincRNA-mRNA pair was chosen. A total of 500 ng RNA was used as a template to synthesize cDNA (10 μL) using the PrimeScript™ RT Master Mix kit (Takara, China). qRT-PCR was performed in a 20 μL reaction volume containing 2 μL of the synthesized cDNA and the SYBR premix Ex-Taq (Takara, DaLian, China) on a qRT-PCR machine (Roche, Switzerland). The PCR amplification program was 94 °C 10 min, followed by 40 cycles of 94 °C for 20 s, 57 °C for 20 s and 72 °C for 20 s. Transcript levels were calculated using the 2^−ΔΔCT^ method, and ACTIN (GenBank Accession number BT013524) was used as a reference gene for gene expression normalization. ACTIN gene-specific primers were 5′- ttgctgaccgtatgagcaag-3′ (forward) and 5′-ggacaatggatggaccagac-3′ (reverse), and the nine transcript specific primers are listed in Additional file 10.

## Additional files

Additional file 1: High-throughput sequencing data and tissue information. (XLSX 11 kb)

Additional file 2: Candidate lincRNA information. (XLSX 2776 kb)

Additional file 3: Candidate ancRNA information. (XLSX 330 kb)

Additional file 4: Candidate sense lncRNA information. (XLSX 33 kb)

Additional file 5: Normalized lincRNA expression values across 3 developmental stages. (XLSX 405 kb)

Additional file 6: Statistical analysis of lincRNA expression levels in the MG versus BR stage. (XLSX 53 kb)

Additional file 7: Statistical analysis of lincRNA expression levels in the MG versus BR + 7 stage. (XLSX 63 kb)

Additional file 8: Statistical analysis of lincRNA expression levels in the BR versus BR + 7 stage. (XLSX 17 kb)

Additional file 9: Information regarding co-regulated lincRNA and mRNA pairs and corresponding correlation. (XLSX 31 kb)

Additional file 10: RT-PCR primer information and relative expression values. (XLSX 107 kb)

## Abbreviations

ancRNAs: antisense non-coding RNAs
BR + 7: Breaker plus seven days
BR: Breaker
DE: Differentially expressed
FC: Fold change
FPKM: Fragment Per Kilobase of exon model per Million mapped reads
lincRNA: long intergenic ncRNAs
lncRNAs: long non-coding RNAs
Mb: Megabase
MG: Mature green
miRNA: microRNA
ncRNAs: non-coding RNAs
PCA: Principal component analysis
piRNA: piwi-interacting RNA
slncRNAs: sense long non-coding RNAs

## References

[1] Lin T, Zhu G, Zhang J, Xu X, Yu Q, Zheng Z, Zhang Z, Lun Y, Li S, Wang X (2014). Genomic analyses provide insights into the history of tomato breeding. Nat Genet.

[2] Kimura S, Sinha N (2008). Tomato (Solanum lycopersicum): a model fruit-bearing crop. CSH protocols.

[3] Giovannoni JJ (2004). Genetic regulation of fruit development and ripening. Plant Cell.

[4] Tomato Genome C: The tomato genome sequence provides insights into fleshy fruit evolution. Nature 2012, 485(7400):635–641. 10.1038/nature11119 PMC3378239 22660326

[5] Matas AJ, Yeats TH, Buda GJ, Zheng Y, Chatterjee S, Tohge T, Ponnala L, Adato A, Aharoni A, Stark R (2011). Tissue- and cell-type specific transcriptome profiling of expanding tomato fruit provides insights into metabolic and regulatory specialization and cuticle formation. Plant Cell.

[6] Butelli E, Titta L, Giorgio M, Mock HP, Matros A, Peterek S, Schijlen EG, Hall RD, Bovy AG, Luo J (2008). Enrichment of tomato fruit with health-promoting anthocyanins by expression of select transcription factors. Nat Biotechnol.

[7] Kumar R, Khurana A, Sharma AK (2014). Role of plant hormones and their interplay in development and ripening of fleshy fruits. J Exp Bot.

[8] Osorio S, Alba R, Damasceno CM, Lopez-Casado G, Lohse M, Zanor MI, Tohge T, Usadel B, Rose JK, Fei Z (2011). Systems biology of tomato fruit development: combined transcript, protein, and metabolite analysis of tomato transcription factor (nor, rin) and ethylene receptor (Nr) mutants reveals novel regulatory interactions. Plant Physiol.

[9] Martel C, Vrebalov J, Tafelmeyer P, Giovannoni JJ (2011). The tomato MADS-box transcription factor RIPENING INHIBITOR interacts with promoters involved in numerous ripening processes in a COLORLESS NONRIPENING-dependent manner. Plant Physiol.

[10] Shinozaki Y, Nicolas P, Fernandez-Pozo N, Ma Q, Evanich DJ, Shi Y, Xu Y, Zheng Y, Snyder SI, Martin LBB (2018). High-resolution spatiotemporal transcriptome mapping of tomato fruit development and ripening. Nat Commun.

[11] Kumar V, Irfan M, Ghosh S, Chakraborty N, Chakraborty S, Datta A. Fruit ripening mutants reveal cell metabolism and redox state during ripening. Protoplasma. 2015; 10.1007/s00709-015-0836-z 26008650

[12] Zhu M, Chen G, Zhou S, Tu Y, Wang Y, Dong T, Hu Z (2014). A new tomato NAC (NAM/ATAF1/2/CUC2) transcription factor, SlNAC4, functions as a positive regulator of fruit ripening and carotenoid accumulation. Plant & cell physiology.

[13] Vrebalov J, Ruezinsky D, Padmanabhan V, White R, Medrano D, Drake R, Schuch W, Giovannoni J (2002). A MADS-box gene necessary for fruit ripening at the tomato ripening-inhibitor (rin) locus. Science.

[14] Barry CS, McQuinn RP, Chung MY, Besuden A, Giovannoni JJ (2008). Amino acid substitutions in homologs of the STAY-GREEN protein are responsible for the green-flesh and chlorophyll retainer mutations of tomato and pepper. Plant Physiol.

[15] Giovannoni JJ (2007). Fruit ripening mutants yield insights into ripening control. Curr Opin Plant Biol.

[16] Manning K, Tor M, Poole M, Hong Y, Thompson AJ, King GJ, Giovannoni JJ, Seymour GB (2006). A naturally occurring epigenetic mutation in a gene encoding an SBP-box transcription factor inhibits tomato fruit ripening. Nat Genet.

[17] Klee HJ, Giovannoni JJ (2011). Genetics and control of tomato fruit ripening and quality attributes. Annu Rev Genet.

[18] Li L, Zhu B, Fu D, Luo Y (2011). RIN transcription factor plays an important role in ethylene biosynthesis of tomato fruit ripening. J Sci Food Agric.

[19] Liu R, How-Kit A, Stammitti L, Teyssier E, Rolin D, Mortain-Bertrand A, Halle S, Liu M, Kong J, Wu C (2015). A DEMETER-like DNA demethylase governs tomato fruit ripening. Proc Natl Acad Sci U S A.

[20] Johnson JM, Edwards S, Shoemaker D, Schadt EE (2005). Dark matter in the genome: evidence of widespread transcription detected by microarray tiling experiments. Trends in genetics : TIG.

[21] Kapranov P, Cheng J, Dike S, Nix DA, Duttagupta R, Willingham AT, Stadler PF, Hertel J, Hackermuller J, Hofacker IL (2007). RNA maps reveal new RNA classes and a possible function for pervasive transcription. Science.

[22] Ng SY, Lin L, Soh BS, Stanton LW (2013). Long noncoding RNAs in development and disease of the central nervous system. Trends in genetics : TIG.

[23] Liu J, Wang H, Chua NH (2015). Long noncoding RNA transcriptome of plants. Plant Biotechnol J.

[24] Zhang X, Zou Z, Zhang J, Zhang Y, Han Q, Hu T, Xu X, Liu H, Li H, Ye Z (2011). Over-expression of sly-miR156a in tomato results in multiple vegetative and reproductive trait alterations and partial phenocopy of the sft mutant. FEBS Lett.

[25] Wu G, Park MY, Conway SR, Wang JW, Weigel D, Poethig RS (2009). The sequential action of miR156 and miR172 regulates developmental timing in Arabidopsis. Cell.

[26] Bartel DP (2009). MicroRNAs: target recognition and regulatory functions. Cell.

[27] Shukla GC, Singh J, Barik S (2011). MicroRNAs: processing, maturation, target recognition and regulatory functions. Mol Cell Pharmacol.

[28] Xin C, Liu W, Lin Q, Zhang X, Cui P, Li F, Zhang G, Pan L, Al-Amer A, Mei H (2015). Profiling microRNA expression during multi-staged date palm (Phoenix dactylifera L.) fruit development. Genomics.

[29] Karlova R, van Haarst JC, Maliepaard C, van de Geest H, Bovy AG, Lammers M, Angenent GC, de Maagd RA (2013). Identification of microRNA targets in tomato fruit development using high-throughput sequencing and degradome analysis. J Exp Bot.

[30] Moxon S, Jing R, Szittya G, Schwach F, Rusholme Pilcher RL, Moulton V, Dalmay T (2008). Deep sequencing of tomato short RNAs identifies microRNAs targeting genes involved in fruit ripening. Genome Res.

[31] Lam P, Zhao L, Eveleigh N, Yu Y, Chen X, Kunst L (2015). The exosome and trans-acting small interfering RNAs regulate cuticular wax biosynthesis during Arabidopsis inflorescence stem development. Plant Physiol.

[32] Nam JW, Bartel DP (2012). Long noncoding RNAs in C. Elegans. Genome Res.

[33] Zhang YC, Liao JY, Li ZY, Yu Y, Zhang JP, Li QF, Qu LH, Shu WS, Chen YQ (2014). Genome-wide screening and functional analysis identify a large number of long noncoding RNAs involved in the sexual reproduction of rice. Genome Biol.

[34] Kim ED, Sung S (2012). Long noncoding RNA: unveiling hidden layer of gene regulatory networks. Trends Plant Sci.

[35] Bohmdorfer G, Wierzbicki AT (2015). Control of chromatin structure by long noncoding RNA. Trends Cell Biol.

[36] Di C, Yuan J, Wu Y, Li J, Lin H, Hu L, Zhang T, Qi Y, Gerstein MB, Guo Y (2014). **Characterization of stress-responsive lncRNAs in Arabidopsis thaliana by integrating expression, epigenetic and structural features**. The Plant journal : for cell and molecular biology.

[37] Shuai P, Liang D, Tang S, Zhang Z, Ye CY, Su Y, Xia X, Yin W (2014). Genome-wide identification and functional prediction of novel and drought-responsive lincRNAs in Populus trichocarpa. J Exp Bot.

[38] Zhu QH, Stephen S, Taylor J, Helliwell CA, Wang MB (2014). Long noncoding RNAs responsive to fusarium oxysporum infection in Arabidopsis thaliana. The New phytologist.

[39] Li L, Eichten SR, Shimizu R, Petsch K, Yeh CT, Wu W, Chettoor AM, Givan SA, Cole RA, Fowler JE (2014). Genome-wide discovery and characterization of maize long non-coding RNAs. Genome Biol.

[40] Liu J, Jung C, Xu J, Wang H, Deng S, Bernad L, Arenas-Huertero C, Chua NH (2012). Genome-wide analysis uncovers regulation of long intergenic noncoding RNAs in Arabidopsis. Plant Cell.

[41] Wang J, Yu W, Yang Y, Li X, Chen T, Liu T, Ma N, Yang X, Liu R, Zhang B (2015). Genome-wide analysis of tomato long non-coding RNAs and identification as endogenous target mimic for microRNA in response to TYLCV infection. Sci Rep.

[42] Zhu B, Yang Y, Li R, Fu D, Wen L, Luo Y, Zhu H (2015). RNA sequencing and functional analysis implicate the regulatory role of long non-coding RNAs in tomato fruit ripening. J Exp Bot.

[43] Wang X, Ai G, Zhang C, Cui L, Wang J, Li H, Zhang J, Ye Z (2016). Expression and diversification analysis reveals transposable elements play important roles in the origin of Lycopersicon-specific lncRNAs in tomato. The New phytologist.

[44] Cui J, Luan Y, Jiang N, Bao H, Meng J (2017). Comparative transcriptome analysis between resistant and susceptible tomato allows the identification of lncRNA16397 conferring resistance to Phytophthora infestans by co-expressing glutaredoxin. The Plant journal : for cell and molecular biology.

[45] Wang H, Chung PJ, Liu J, Jang IC, Kean MJ, Xu J, Chua NH (2014). Genome-wide identification of long noncoding natural antisense transcripts and their responses to light in Arabidopsis. Genome Res.

[46] Rose JK, Lee HH, Bennett AB (1997). Expression of a divergent expansin gene is fruit-specific and ripening-regulated. Proc Natl Acad Sci U S A.

[47] Zhong S, Fei Z, Chen YR, Zheng Y, Huang M, Vrebalov J, McQuinn R, Gapper N, Liu B, Xiang J (2013). Single-base resolution methylomes of tomato fruit development reveal epigenome modifications associated with ripening. Nat Biotechnol.

[48] Stroud H, Ding B, Simon SA, Feng S, Bellizzi M, Pellegrini M, Wang GL, Meyers BC, Jacobsen SE (2013). Plants regenerated from tissue culture contain stable epigenome changes in rice. elife.

[49] Xin M, Wang Y, Yao Y, Song N, Hu Z, Qin D, Xie C, Peng H, Ni Z, Sun Q (2011). Identification and characterization of wheat long non-protein coding RNAs responsive to powdery mildew infection and heat stress by using microarray analysis and SBS sequencing. BMC Plant Biol.

[50] Pauli A, Valen E, Lin MF, Garber M, Vastenhouw NL, Levin JZ, Fan L, Sandelin A, Rinn JL, Regev A (2012). Systematic identification of long noncoding RNAs expressed during zebrafish embryogenesis. Genome Res.

[51] Pattison RJ, Csukasi F, Zheng Y, Fei Z, van der Knaap E, Catala C (2015). Comprehensive tissue-specific transcriptome analysis reveals distinct regulatory programs during early tomato fruit development. Plant Physiol.

[52] Liu TT, Zhu D, Chen W, Deng W, He H, He G, Bai B, Qi Y, Chen R, Deng XW (2013). A global identification and analysis of small nucleolar RNAs and possible intermediate-sized non-coding RNAs in Oryza sativa. Mol Plant.

[53] Wang Y, Wang X, Deng W, Fan X, Liu TT, He G, Chen R, Terzaghi W, Zhu D, Deng XW (2014). Genomic features and regulatory roles of intermediate-sized non-coding RNAs in Arabidopsis. Mol Plant.

[54] Boerner S, McGinnis KM (2012). Computational identification and functional predictions of long noncoding RNA in Zea mays. PLoS One.

[55] Chen X, Zhou DX (2013). Rice epigenomics and epigenetics: challenges and opportunities. Curr Opin Plant Biol.

[56] Dong R, Jia D, Xue P, Cui X, Li K, Zheng S, He X, Dong K (2014). Genome-wide analysis of long noncoding RNA (lncRNA) expression in hepatoblastoma tissues. PLoS One.

[57] Xing MQ, Zhang YJ, Zhou SR, Hu WY, Wu XT, Ye YJ, Wu XX, Xiao YP, Li X, Xue HW (2015). Global analysis reveals the crucial roles of DNA methylation during Rice seed development. Plant Physiol.

[58] Liu X, Hao L, Li D, Zhu L, Hu S (2015). Long non-coding RNAs and their biological roles in plants. Genomics Proteomics Bioinformatics.

[59] Trapnell C, Pachter L, Salzberg SL (2009). TopHat: discovering splice junctions with RNA-Seq. Bioinformatics.

[60] Trapnell C, Williams BA, Pertea G, Mortazavi A, Kwan G, van Baren MJ, Salzberg SL, Wold BJ, Pachter L (2010). Transcript assembly and quantification by RNA-Seq reveals unannotated transcripts and isoform switching during cell differentiation. Nat Biotechnol.

[61] Quinlan AR, Hall IM (2010). BEDTools: a flexible suite of utilities for comparing genomic features. Bioinformatics.

[62] Krueger F, Andrews SR (2011). Bismark: a flexible aligner and methylation caller for bisulfite-Seq applications. Bioinformatics.

[63] Langmead B, Salzberg SL (2012). Fast gapped-read alignment with bowtie 2. Nat Methods.

